# Differential clinical and prognostic impact of myeloid sarcoma vs medullary myeloid blast phase of chronic myelogenous leukemia in the era of tyrosine kinase inhibitor therapy

**DOI:** 10.1038/bcj.2016.27

**Published:** 2016-05-06

**Authors:** Z Chen, W Wang, J E Cortes, E Liu, R N Miranda, C Zhao, J Yuan, X Lu, W Yang, M D Ameri, H M Kantarjian, L J Medeiros, S Hu

**Affiliations:** 1 Department of Hematopathology, The University of Texas MD Anderson Cancer Center, Houston, TX, USA; 2 Department of Hematology, Huashan Hospital, Fudan University, Shanghai, China; 3 Department of Leukemia, The University of Texas MD Anderson Cancer Center, Houston, TX, USA; 4 Department of Pathology, Institute of Hematology & Blood Diseases Hospital, Tianjin, China; 5 Department of Pathology, University of Iowa, Iowa City, IA, USA; 6 Department of Pathology, University of Nebraska, Omaha, NE, USA; 7 Department of Pathology, City of Hope National Medical Center, Duarte, CA, USA

Myeloid sarcoma (MS) is a rare neoplasm consisting of immature myeloid cells that forms a tumor mass at an anatomic site other than the bone marrow (BM).^1^ MS may develop *de novo* or secondary to other types of myeloid neoplasm. MS is most often associated with acute myeloid leukemia (AML), and develops in 2–5% of AML patients.^2, 3^ Less frequently, MS can arise in patients with chronic myelogenous leukemia (CML), mainly in the setting of blast phase (BP) or accelerated phase (AP).^3, 4, 5^ Traditionally, MS is considered the equivalent of AML, and is one of diagnostic criteria for CML-BP, regardless of the blast count in the BM or peripheral blood. According to the 2008 World Health Organization criteria, CML-BP can be diagnosed when: (1) blasts are ⩾20% in the BM or peripheral blood (medullary BP); or (2) there is an extramedullary blast proliferation, in other words, MS.

In the era of pre-tyrosine kinase inhibitor (TKI) therapy, most cases of chronic myelogenous leukemia (CML) progressed to blast phase (BP) within 2–3 years after the initial diagnosis of CML, chronic phase (CP). Approximately 7–17% of patients with CML-BP developed myeloid sarcoma (MS).^5, 6^ The median survival of CML patients with MS was 3–6 months, comparable with that of patients with CML in medullary BP. With TKIs becoming the standard and front-line therapy, the risk of blastic transformation has been greatly reduced.^7, 8^ The long-term cumulative probability of progression to BP is only ~5%.^7, 9^ Correspondingly, as a sign of progression of CML, MS has become increasingly less common. In literature, MS evolving in CML patients in the era of TKI therapy is only rarely reported, mostly in the form of single-case reports, and thus the prognostic impact of MS in CML patients has not been studied systematically. It remains unknown whether MS and medullary BP confer similar clinical and prognostic value in the era of TKI therapy.

We studied 307 CML patients: 42 had extramedullary MS and a history of or concurrent CML in the bone marrow (BM), and 265 had medullary myeloid BP (MyBP) but without previous or concurrent MS. All cases of MS and MyBP were diagnosed from 2000 to 2015, and the diagnoses were confirmed by histopathology and ancillary studies (Figures 1a–c). MyBP was defined to have ⩾20% myeloblasts in the BM or peripheral blood. If MS or MyBP was present at initial diagnosis of CML, they were designated as MS1 or MyBP1, respectively. If MS or MyBP developed later during the course of treatment, the disease was designated as MS2 or MyBP2, respectively. The overall survival (OS) was calculated from the date of diagnosis of MS or MyBP to the date of last follow-up or death.

In the MS group, there were 34 men and 8 women with a male-to-female ratio of 4.3:1 (Table 1 and Supplementary Table 1). The median age was 49.2 years at diagnosis of MS (range, 19.4–82.7 years). The median interval from the initial diagnosis of CML to the diagnosis of MS was 18.3 months (range, 0–305.8 months). Thirty-eight patients (90.5%) received TKI therapy, 33 (78.6%) received chemotherapy and 8 received allogeneic hematopoietic stem cell transplantation. These patients were further stratified into three subgroups based on the time when MS emerged and the blast counts in the BM: 13 had MS1 with the concurrent BM in CML-CP (MS1+CP), 17 had MS2 with the concurrent BM in CML-CP (MS2+CP) and 12 had MS2 with the concurrent BM in MyBP (MS2+MyBP2) (Figures 1a–c). No MS1 case was identified with the concurrent BM in MyBP. MS commonly involved skin (16/42, 38.1%), bone (11/42, 26.2%) and lymph nodes (10/42, 23.8%), and often involved multiple anatomic sites. MS2 was more frequently multifocal than MS1 (7/13, 53.8% for MS1; 27/29, 93.1% for MS2, *P*=0.006). The detailed treatment regimens, cytogenetic profiles, *ABL* mutation status and outcome, including initial response of both MS and medullary disease, disease status at last follow-up and causes of death are listed in Table 1 and Supplementary Tables 1 and 2.

In the MyBP group, there were 154 men and 111 women with a male-to-female ratio of 1.4:1. The median age was 52.4 years at diagnosis of MyBP (range, 15.4–92.4 years). The median interval from the initial diagnosis of CML to the diagnosis of MyBP was 25.4 months (range, 0–232.1 months). Similarly, the MyBP patients were further stratified into two subgroups based on the time of blastic transformation: 23 had MyBP1 and 242 had MyBP2.

As MS is considered the equivalent of CML-BP, we first examined whether MS and MyBP were prognostically similar in patient survival. As shown in Figure 1d, patients with MS had a much better OS than those with MyBP (median OS: 18.4 months and 8.0 months, respectively, *P*=0.01).

We next examined whether the time and the BM blast count when MS developed affect survival of CML patients with MS. As shown in Figure 1e, patients with MS1+CP had significantly better OS than the patients with MS2, regardless of the BM blast counts (MS1+CP vs MS2+CP, *P*=0.002; MS1+CP vs MS2+MyBP, *P*=0.0006). However, there was no difference in survival between patients with MS2+CP vs those with MS2+MyBP2 (*P*=0.60). The median survivals for the patients with MS1+CP, MS2+CP and MS2+MyBP2 were 36.0, 8.3 and 8.7 months, respectively.

We also compared the prognostic impact of MS vs MyBP based on the time when MS or MyBP develop. As shown in Figure 1f, patients with MyBP1 had significantly better survival than those with MyBP2 (median survival: 17.5 and 7.0 months, respectively, *P*=0.02). Furthermore, patients with MS1+CP had significantly more favorable survival than those with MyBP1 (median survival: 36.0 and 17.5 months, respectively, *P*=0.04, Figure 1g). However, patients with MS2+CP had a dismal survival similar to those with MyBP2 (median survival: 8.3 and 7.0 months, respectively, *P*=0.55, Figure 1h).

Given the differential outcome of patients with MS1 vs those with MS2, we then compared these two subgroups of patients for the treatment response of both extramedullary and medullary disease (Table 1 and Supplementary Table 1). The vast majority of MS1 patients (11/12, 91.7%) achieved initial complete remission of MS compared with MS2 patients (13/25, 52.0%, *P*=0.03). Furthermore, the vast majority of MS1 patients achieved initial complete cytogenetic response (CCyR) or deeper remission of medullary disease compared with MS2 patients (10/12, 83.3% for MS1; 10/25, 40.0% for MS2, *P*=0.02). Of those who achieved initial CCyR or deeper response of medullary disease, 1/10 MS1 and 5/10 MS2 patients had relapsed/progressive medullary disease. Of patients with available follow-up information regarding the disease status at last follow-up, 3/13 MS1 and 19/25 MS2 patients (*P*=0.004) died of progressive disease.

We also compared patients with MS1 and those with MyBP1 for the treatment response of medullary disease (Table 1 and Supplementary Table 3). Less than half of MyBP1 patients achieved CCyR or deeper remission of medullary disease compared with MS1 patients (11/23, 47.8% for MyBP1; 10/12, 83.3% for MS1, *P*=0.04). Twelve of 23 MyBP1 patients died of progressive disease compared with 3/13 MS1 patients (*P*=0.09).

Here we characterized the clinical features of MS in 42 CML patients. The median age of patients at diagnosis of MS was ~49 years, 3 years younger than that of patients with MyBP. The median interval from the initial diagnosis of CML to the diagnosis of MS was ~20 months, 5 months shorter than that of MyBP transformation. The male-to-female ratio for MS patients was 4.3:1 vs 1.4:1 for MyBP patients, the latter being similar to that in our entire cohort of CML.^10, 11^ Interestingly, MS was multifocal in almost all MS2 patients compared with in about half of MS1 patients.

The evidence of supporting the use of MS as a criterion for CML-BP was derived mainly from the pre-TKI era;^5, 6, 12^ the prognostic impact of MS in the TKI era was largely unknown. In our study, the median survival of patients with MyBP1 and MyBP2 was ~17.5 and 7.0 months, respectively. Patients with MS2 had a median survival of ~8.0 months, similar to that in patients with MyBP2. In contrast, patients with MS1 had a median survival of 36.0 months. Correspondingly, patients with MS1 more likely achieved complete remission of MS, and CCyR or deeper remission of medullary disease, suggesting that MS as the initial manifestation of CML may be biologically different from MS or medullary MyBP as the late sequela, which indicates treatment resistance and disease progression. Interestingly, patients with MS1 also had more favorable outcome than those with MyBP1. Given that the survival of this subgroup of patients appears closer to that of patients with CML in AP,^13, 14, 15^ it will probably be better to categorize this subgroup of cases in AP instead of BP from a prognostic standpoint although the better outcome itself does not dictate treatment regimens or a change of treatment.

In summary, as one sign of CML progression, MS has become increasingly less common in patients with CML in the era of TKI therapy. MS and medullary MyBP may confer different clinical and prognostic significance related, in part, to the time of presentation. Although MS and medullary MyBP arising late during the course of treatment confer a similarly dismal prognosis, patients with MS without medullary BP at initial diagnosis had a significantly more favorable outcome and counted for the overall better prognosis of patients with MS than those with CML in medullary MyBP. To the best of our knowledge, this study is the largest and also the first case series on MS in CML patients in the era TKI therapy.

## Figures and Tables

**Figure 1:**
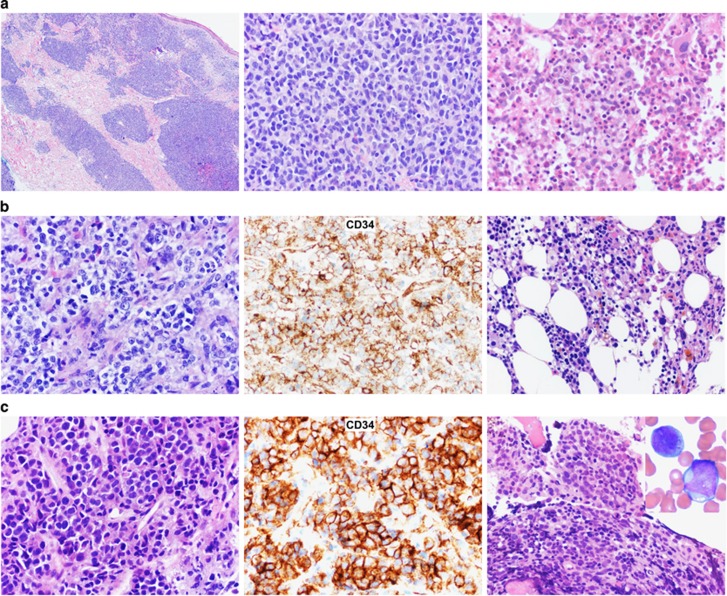

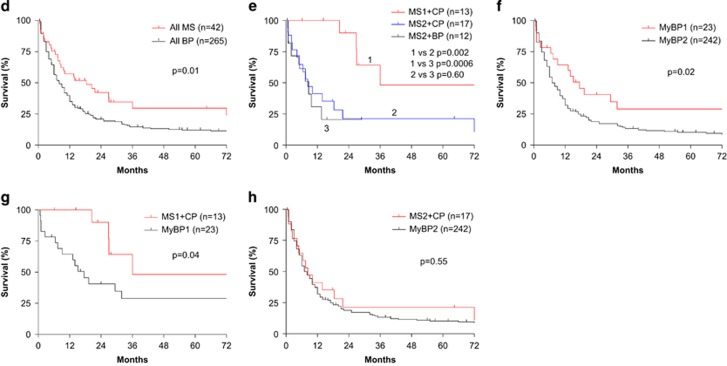
Morphology and prognostic impact of myeloid sarcoma vs medullary myeloblast phase of CML. (**a**) A representative case of MS1+CP: the patient had concurrent MS of skin (left and middle panels) and CML, medullary CP (right panel) as the initial manifestations. (**b**) A representative case of MS2+CP: the patient had concurrent MS of left tibia (left and middle panels) and CML, medullary CP (right panel) as the late manifestations, 24 months after initial diagnosis of CML-CP. (**c**) A representative case of MS2+MyBP2: the patient had concurrent MS of right hip (left and middle panels) and CML, medullary MyBP (right panel), 3 months after initial diagnosis of CML-CP. (**d**) Survival comparison between all CML patients with MS and all patients with medullary MyBP. (**e**) Survival comparison between subgroups of CML patients with MS arising at different time course of CML treatment. (**f**) Survival comparison between CML patients with MyBP1 vs patients with MyBP2. (**g**) Survival comparison between CML patients with MS1+CP vs patients with MyBP1. (**h**) Survival comparison between CML patients with MS2+CP vs patients with MyBP2. Of these 42 MS patients, 36 were diagnosed and treated at the University of Texas MD Anderson Cancer Center (MDACC) and the remaining 6 in other institutions. All MyBP patients without MS were diagnosed and treated at MDACC. Cases of CML-BP with lymphoblastic or mixed immunophenotype were not included. Survival curves were built using the Kaplan–Meier method, and the differences in survival between subgroups were analyzed by the log-rank test. The study is approved by the Institutional Review Board MDACC. Abbreviations: MyBP1, MyBP at initial diagnosis of CML; MyBP2, MyBP arising later during treatment of CML; MS1, MS at initial diagnosis of CML; MS2, MS arising later during treatment of CML.

**Table 1:** Treatment and outcome of 42 CML patients with myeloid sarcoma

*No.*	*Sex*	*Age*	*MS subgroups*	*Post-MS treatment*	*Response of MS*^a^	*Response of CML*^a^	*F/U time*^b^ *(months)*	*Status at last F/U*	*Cause of Death*	*ABL mutations*
1	M	72.7	MS1-CP	TKI	PR	SD	20.5	Dead	Leukemia PD, MS PD	No^c^
2	M	47.8	MS1-CP	CT+TKI+RT	CR	SD	26.9	Dead	Leukemia PD	NT
3	M	50.0	MS1-CP	CT+TKI Allo-HSCT	CR	MMR	27.0	Dead	GvHD	NT
4	M	52.3	MS1-CP	CT+TKI	CR	CCyR	36.0	Dead	Leukemia PD, MS PD	T315I^c^
5	M	30.3	MS1-CP	CT+TKI	NA	NA	6.2	Alive		NT
6	F	63.5	MS1-CP	TKI	CR	MMR	14.4	Alive		NT
7	M	30.7	MS1-CP	TKI	CR	CCyR	14.3	Alive		NT
8	M	72.8	MS1-CP	TKI	CR	MMR	22.8	Alive		NT
9	M	29.2	MS1-CP	CT+TKI	CR	CMR	22.8	Alive		NT
10	M	52.7	MS1-CP	CT+TKI	CR	MMR	27.8	Alive		NT
11	F	23.3	MS1-CP	CT+TKI Allo-HSCT	CR	CMR	118.8	Alive		NT
12	M	52.7	MS1-CP	CT+TKI Allo-HSCT	CR	CMR	140.9	Alive		NT
13	M	45.3	MS1-CP	CT+TKI+RT	CR	CMR	146.6	Alive		No^c^
14	M	36.3	MS2-CP^d^	CT+TKI	PD	PD	0.8	Dead	Leukemia PD, MS PD	No
15	M	26.8	MS2-CP	CT+TKI	PD	PD	0.9	Dead	Leukemia PD, MS PD	NT
16	M	75.1	MS2-CP	TKI	PR	PD	2.1	Dead	Leukemia PD	NT
17	M	51.0	MS2-CP^d^	CT+TKI+RT	PR	PD	2.4	Dead	Leukemia PD	No
18	M	23.2	MS2-CP	CT+TKI	PR	PD	4.2	Dead	Leukemia PD, MS PD	No
19	M	47.8	MS2-CP^d^	CT+TKI Allo-HSCT	CR	CCyR	4.9	Dead	Leukemia PD	T315I
20	M	23.5	MS2-CP	CT+TKI	CR	CHR	6.5	Dead	Leukemia PD	E255V
21	M	55.5	MS2-CP	CT+TKI	CR	CCyR	7.5	Dead	Leukemia PD, MS PD	NT
22	F	51.7	MS2-CP	CT	NA	NA	8.3	Dead	NA	NT
23	M	51.3	MS2-CP	CT+TKI	PR	SD	10.1	Dead	Leukemia PD, MS PD	NT
24	M	43.1	MS2-CP	CT+TKI+RT	PR	SD	13.9	Dead	Leukemia PD	E255K
25	M	51.4	MS2-CP	TKI+RT	CR	CCyR	18.4	Dead	Leukemia PD, MS PD	NT
26	F	48.4	MS2-CP^d^	TKI	CR	MMR	21.7	Dead	Leukemia PD,	NT
27	M	57.3	MS2-CP	CT+TKI Allo-HSCT	CR	CMR	88.5	Dead	Esophageal cancer	No
28	F	39.2	MS2-CP	CT+TKI	CR	CHR	17.8	Alive		NT
29	F	58.4	MS2-CP	TKI	CR	MMR	64.4	Alive		NT
30	M	56.1	MS2-CP	TKI	CR	CMR	126.9	Alive		NT
31	M	19.4	MS2-BP	CT+TKI	PD	PD	0.8	Dead	Leukemia PD	E255V/K
32	M	82.7	MS2-BP	CT+TKI	PD	PD	2.0	Dead	Leukemia PD	NT
33	M	45.8	MS2-BP	CT+TKI	PD	PD	0.6	Dead	Leukemia PD, MS PD	No
34	F	31.9	MS2-BP	CT+TKI+RT Allo-HSCT	PR	CHR	5.2	Dead	Leukemia PD	No
35	M	53.4	MS2-BP	CT+TKI	PD	PD	7.4	Dead	Leukemia PD	NT
36	M	53.2	MS2-BP	CT+TKI	CR	PD	8.7	Dead	Leukemia PD	F317L
37	M	54.3	MS2-BP	CT+TKI Allo-HSCT	CR	CCyR	9.7	Dead	Leukemia PD	No
38	M	34.9	MS2-BP	CT+TKI	CR	MMR	13.6	Dead	Infection	E255
39	M	38.4	MS2-BP	CT	NA	NA	15.7	Alive		NT
40	M	22.0	MS2-BP	CT	NA	NA	0.5	Alive^e^		NT
41	F	59.0	MS2-BP	CT	NA	NA	1.0	Alive^e^		NT
42	M	44.3	MS2-BP	CT+TKI Allo-HSCT	CR	MMR	28.9	Alive		NT

Abbreviations: age, age at diagnosis of MS; allo-HSCT, allogeneic hematopoietic stem cell transplantation; BP, blast phase; CCyR, complete cytogenetic response; CHR, complete hematologic response; CML, chronic myelogenous leukemia; CP, chronic phase; CR, complete response; CT, chemotherapy; F, female; F/U, follow-up; GvHD, graft-vs-host disease; M, male; MMR, major molecular response; MS, myeloid sarcoma; MS1, MS at initial diagnosis of CML; MS2, MS arising during course of treatment; NA, not applicable; NT, not tested; PD, progressive disease; PR, partial remission; RT, radiation treatment; SD, stable disease; TKI, tyrosine kinase inhibitor. CR of MS was defined as complete disappearance of the lesion(s). PR of MS was defined as decreased size but not complete disappearance of the lesion(s). CHR, CCyR and MMR are defined according to the NCCN and European LeukemiaNet Guidelines.

a The best initial response of extramedullary and medullary disease after MS emergence.

b The follow-up time after MS emergence.

c ABL mutations detected after MS emergence.

d The patients had a history of CML-BP but reverted to CP status when MS2 developed.

e Lost F/U after one course of treatment.
