# Supplementary material for: Differential clinical and prognostic impact of myeloid sarcoma vs medullary myeloid blast phase of chronic myelogenous leukemia in the era of tyrosine kinase inhibitor therapy
Source: Blood Cancer J. 2016 May 6;6(5):e418–. doi: 10.1038/bcj.2016.27 (PMC4916296; doi:10.1038/bcj.2016.27)
Supplement: Supplementary Tables [file bcj201627x1.docx]

| **Supplementary Table 1 Overall clinical features of 42 CML patients with myeloid sarcoma** | | | | |
| --- | --- | --- | --- | --- |
|  | **All MS  (n=42)** | **MS1+medullary CP  (n=13)** | **MS2+medullary CP  (n=17)** | **MS2+medullary BP  (n=12)** |
| **Sex** |  |  |  |  |
| Male | 34 | 11 | 13 | 10 |
| Female | 8 | 2 | 4 | 2 |
| **Age** |  |  |  |  |
| Median | 49.2 | 50.0 | 51.0 | 45.1 |
| Range | 19.4~82.7 | 23.3~72.8 | 23.2~75.1 | 19.4~82.7 |
| **Sites** |  |  |  |  |
| Skin | 16 | 6 | 5 | 5 |
| Bone | 11 | 3 | 7 | 1 |
| Lymph nodes | 10 | 3 | 1 | 6 |
| Abdomen/pelvis | 2 | 1 | 1 | 0 |
| Oral cavity | 1 | 0 | 1 | 0 |
| Testis | 1 | 0 | 1 | 0 |
| Uterus | 1 | 0 | 1 | 0 |
| **Foci** |  |  |  |  |
| Single | 8 | 6 | 2 | 0 |
| Multiple | 34 | 7 | 15 | 12 |
| **Interval** |  |  |  |  |
| Median | 18.3 | 0 | 43.7 | 28.4 |
| Range | 0~305.8 | 0 | 4.7~305.8 | 2.6~121.4 |
| **Treatment** |  |  |  |  |
| TKI | 9 | 4 | 5 | 0 |
| Chemotherapy | 4 | 0 | 1 | 3 |
| Chemotherapy + TKI | 29 | 9 | 11 | 9 |
| Allo-HSCT | 8 | 3 | 2 | 3 |
| **MS Response** |  |  |  |  |
| PD | 6 | 0 | 2 | 4 |
| PR | 7 | 1 | 5 | 1 |
| CR | 24 | 11 | 9 | 4 |
| NA | 5 | 1 | 1 | 3 |
| **Medullary response** |  |  |  |  |
| PD | 10 | 0 | 5 | 5 |
| SD | 4 | 2 | 2 | 0 |
| CHR | 3 | 0 | 2 | 1 |
| CCyR | 6 | 2 | 3 | 1 |
| >= MMR | 14 | 8 | 4 | 2 |
| NA | 5 | 1 | 1 | 3 |

Abbreviations: CML: chronic myelogenous leukemia; MS: myeloid sarcoma; MS1: MS at initial diagnosis of CML; MS2: MS arising during course of treatment; CP: chronic phase; BP: blast phase; Interval: interval time from initial diagnosis of CML to emergence of MS; TKI: tyrosine kinase inhibitor; CT: chemotherapy; RT: radiation treatment; Allo-HSCT: allogeneic hematopoietic stem cell transplantation; PD: progressive disease; SD: stable disease; CR: complete response; PR: partial remission; CHR: complete hematologic response; CCyR: complete cytogenetic response; MMR: major molecular response. CR of MS was defined as complete disappearance of the lesion(s). PR of MS was defined as decreased size but not complete disappearance of the lesion(s). CHR, CCyR and MMR are defined according to the NCCN and European LeukemiaNet Guidelines.

**Supplementary Table 2 Karyotypes of bone marrow at diagnosis of myeloid sarcoma**

| **No.** | **Karyotype** |
| --- | --- |
| 1 | 46,XY,der(7)t(7;11)(p12;p11.2)add(7)(q35),der(11)del(11)(p11.2)t(11;?)(p11.2;?)[14]/46,XY,der(7)t(7;11)(p12;p11.2)add(7)(q35),t(9;22)(q34;q11.2),der(11)del(11)(p11.2)t(11;?)(p11.2;?)[3]/46,XY[3] |
| 2 | 46,XY,t(9;22)(q34;q11.2) [20] |
| 3 | 46,XY,t(9;22)(q34;q11.2)[20] |
| 4 | 46,XY,t(9;22)(q34;q11.2) [7]/46,XY,t(9;22)(q34;q11.2) +9[13] |
| 5 | 46,XY,t(9;22)(q34;q11.2)[4]/46,sl,inv(3)(q21q26.2)[6]/46,sdl1,t(1;12)(q21;q13)[5]/46,sdl1,i(17)(q10)[5] |
| 6 | 46,XX,der(9)t(9;22)(q34;q11.2)del(9)(q34),der(22)t(9;22)[20] |
| 7 | 46,XY,t(9;22)(q34;q11.2)[20] |
| 8 | 46,XY,t(9;22)(q34;q11.2)[5]/48,sl,+8,+21[4]/46,XY[11] |
| 9 | NA (complex karyotype) |
| 10 | 46,XY,t(9;22)(q34;q11.2)[19]/47,sl,+mar[1] |
| 11 | 46,XX,t(9;22)(q34;q11.2)[20] |
| 12 | 46,XY,t(3;15)(q27;q15),t(9;22)(q34;q11.2)[20] |
| 13 | 46,XY,t(9;22)(q34;q11.2),-18,+der(22)t(9;22)t(18;22)(q11.2;p13)[4]/47,idem,+8[13]/46,XY[3] |
| 14 | NA (cryptic) |
| 15 | NA (cryptic) |
| 16 | 46,XY,t(9;22)(q34;q11.2)[20] |
| 17 | 46,XY,t(9;22)(q34;q11.2)[10]/49,idem,-7,+8,+8,+19,+der(22)t(9;22)[10] |
| 18 | 48,XY,+8,t(9;22)(q34;q11.2),+19[2]/46,XY[18] |
| 19 | NA (CCyR) |
| 20 | 46,XY,t(9;22)(q34;q11.2)[5]/47,XY,der(9)t(9;22)(q34;q11.2),der(22)idic(22)(p11.2)t(9;22),+der(22)idic(22)(p11.2)t(9;22)[15] |
| 21 | NA (CCyR) |
| 22 | NA (not done) |
| 23 | 46,XY,t(9;22)(q34;q11.2)[3]/46,XY[17] |
| 24 | 46,XY,t(9;22)(q34;q11.2)[3]/47,XY,t(9;22)(q34;q11.2),+19[1]/46,XY[8] |
| 25 | NA (CCyR) |
| 26 | NA (CCyR) |
| 27 | NA (MMR) |
| 28 | 46,XX,t(9;22)(q34;q11.2)[20] |
| 29 | NA (CCyR) |
| 30 | 50,XY,t(1;19)(q12;q13.1),+8,+8,+8,t(9;22)(q34;q11.2),i(17)(q10),+der(19)t(1;19)[20] |
| 31 | 46,XY,add(7)(p22),t(9;22)(q34;q11.2),t(12;15)(q13;q24),del(17)(q21)[8]/46,XY[12] |
| 32 | 45,t(X;9;22)(q25;q34;q11.2),-Y[19]/ 38,der(X)t(X;9;22)(q25;q34;q11.2),-Y,-3,-4,-5,-7,-9,der(11)t(?;9)(?;q34)t(9;11)(q34;p15),-12,-14,-15,-16,-20,-22,der(22)t(X;9;22),+4mar[1] |
| 33 | 46,XY,t(9;11)(p22;q13),t(9;22)(q34;q11.2)[5]/46,XY[15] |
| 34 | 46,XX,t(9;22)(q34;q11.2)[7]/33<2n>,XX,-2,-3,-4,-5,-6,-7,t(9;22)(q34;q11.2),-10,-12,-13,-15,-16,-17,-18[12] |
| 35 | 47,XY,t(9;22)(q34;q11.2),+der(22)t(9;22)[18]/46,XY,der(9)t(9;22)(q34;q11.2),ider(22)(q10)t(9;22)[2] |
| 36 | 46,XY,del(5)(q13q22),add(6)(q27),add(8)(q22),t(9;22)(q34;q11.2)[13]/46,XY,del(5)(q13q22),add(6)(q27),t(9;22)(q34;q11.2)[4] |
| 37 | 48,XY,t(9;22)(q34;q11.2),+19,+der(22)t(9;22)(q34;q11.2)[19] |
| 38 | 49,XY,+8,+8,t(9;22)(q34;q11.2),i(17)(q10),+der(22)t(9;22)[17]/44-48,XY,+8,t(9;22)(q34;q11.2),i(17)(q10),+der(22)t(9;22)[cp3] |
| 39 | NA (not done) |
| 40 | 46,XX,t(9;22)(q34;q11)[4]/46,XY[7] |
| 41 | 46,XX,t(9;22)(q34;q11.2)[6] |
| 42 | NA (not done) |

Abbreviations: NA: not applicable, either because of 1) karyotyping not performed; 2) complete cytogenetic remission or deeper remission; 3) cryptic; or 4) detailed karyotype not provided by the outside institution. CCyR: complete cytogenetic response; MMR: major molecular response. *BCR-ABL1* was positive in all cases by FISH and/or RT-PCR analyses.

**Supplementary Table 3 Treatment and outcome of patients with CML-MyBP1**

| **No.** | **Sex** | **Treatment** | **F/U time (months)** | **Status at last F/U** | **Allo-HSCT** | **Response^a^** | **Cause of death** | **ABL mutations** |
| --- | --- | --- | --- | --- | --- | --- | --- | --- |
| 1 | M | CT | 0.7 | Dead | No | No CHR | Leukemia PD | NT |
| 2 | M | CT+TKI | 6.5 | Dead | No | No CHR | Leukemia PD | T315I |
| 3 | F | TKI | 13.2 | Dead | No | No CHR | Leukemia PD | Y253F |
| 4 | M | CT+TKI | 15.2 | Dead | No | No CHR | Leukemia PD | No |
| 5 | M | CT | 1.0 | Dead | No | No CHR | Leukemia PD | NT |
| 6 | M | CT+TKI | 2.6 | Dead | No | No CHR | Leukemia PD | NT |
| 7 | M | CT+TKI | 9.2 | Dead | No | No CHR | Leukemia PD | NT |
| 8 | M | CT+TKI | 1.0 | Dead | No | No CHR | Leukemia PD | NT |
| 9 | M | CT | 0.9 | Dead | No | No CHR | Leukemia PD | NT |
| 10 | F | CT+TKI | 7.4 | Dead | No | CHR | Leukemia PD | NT |
| 11 | F | CT+TKI | 31.9 | Dead | No | CHR | Leukemia PD | No |
| 12 | M | CT+TKI | 29.4 | Dead | No | CHR | Leukemia PD | L298V |
| 13 | M | CT | 19.3 | Dead | Yes | CMR | Infection | No |
| 14 | F | CT+TKI | 14.0 | Dead | Yes | CCyR | Infection | NT |
| 15 | F | CT+TKI | 17.5 | Dead | Yes | CMR | GVHD | Q252H |
| 16 | F | CT+TKI | 118.5 | Alive | No | MMR | Alive | G254W |
| 17 | F | CT+TKI | 78.0 | Alive | No | MMR | Alive | E255K |
| 18 | M | TKI | 16.1 | Alive | No | MMR | Alive | NT |
| 19 | M | CT+TKI | 5.1 | Alive | No | MMR | Alive | NT |
| 20 | M | CT+TKI | 24 | Alive | Yes | CMR | Alive | NT |
| 21 | M | CT | 90.5 | Alive | Yes | CMR | Alive | NT |
| 22 | F | CT+TKI | 160.1 | Alive | Yes | CMR | Alive | NT |
| 23 | F | CT+TKI | 144.9 | Alive | Yes | CMR | Alive | NT |

Abbreviations: MyBP1: myeloid blast phase as the initial presentation of CML; TKI: tyrosine kinase inhibitor; CT: chemotherapy; Allo-HSCT: allogeneic hematopoietic stem cell transplantation; F/U: follow up; PD: progressive disease; CHR: complete hematologic response; CCyR: complete cytogenetic response; MMR: major molecular response; CMR: complete molecular response;.GVHD: graft-versus-host-disease; NT: not tested. CHR, CCyR, MMR and CMR are defined according to the NCCN and European LeukemiaNet Guidelines.

^a^ The best initial response to treatment.
